# The effect of renal impairment and obesity on anti-Xa peak and trough levels in patients receiving therapeutic doses of nadroparin: a comparison with control patients

**DOI:** 10.1007/s00228-023-03558-5

**Published:** 2023-09-14

**Authors:** L. Mast, M. Y. M. Peeters, M. Söhne, C. M. Hackeng, C. A. J. Knibbe, M. P. H. van den Broek

**Affiliations:** 1 Department of Clinical Pharmacy, Zaandam Medical Centre, Zaandam, The Netherlands; 2 Department of Clinical Pharmacy, St. Antonius Hospital, Nieuwegein, The Netherlands; 3 Department of Hematology, St. Antonius Hospital, Nieuwegein, The Netherlands; 4 Department of Clinical Chemistry, St. Antonius Hospital, Nieuwegein, The Netherlands; 5 Division of Systems Biomedicine & Pharmacology, Leiden Academic Centre for Drug Research, Leiden University, Leiden, The Netherlands; 6 Department of Pharmaceutical Sciences, Utrecht University, Utrecht, The Netherlands

**Keywords:** LMWH, Nadroparin, Anti-Xa, Renal impairment, Obesity, Dose reduction

## Abstract

**Purpose:**

Anti-Xa peak level monitoring is recommended during LMWH treatment in renal impairment or obesity. The trough level has been proposed as marker for bleeding. We studied the influence of renal impairment and obesity on anti-Xa levels.

**Methods:**

Peak and trough levels were collected during therapeutic nadroparin treatment in patients with renal impairment, obese patients, and controls. 27 patients (*n* = 68 samples) were evaluated and combined with published data (*n* = 319 samples from 35 patients) using population pharmacokinetic (popPK) modelling.

**Results:**

Median peak level was 0.44 and 0.95 IU/mL in renal impairment with and without dose reduction and 0.60 and 0.43 IU/mL in obesity and controls, respectively. Trough levels were < 0.5 IU/mL in all patients with renal impairment with dose reduction and in 5/6 control patients. In the popPK model, total body weight and eGFR were covariates for clearance and lean body weight for distribution volume. Model-based evaluations demonstrated peak levels below the therapeutic window in controls and increased levels in renal impairment. Dose reductions resulted in a different effect on peak and trough levels. Obese patients (BMI up to 32 kg/m^2^) had similar levels upon weight-based dosing.

**Conclusion:**

In renal impairment, anti-Xa peak levels after dose reduction are comparable to those in controls. Weight-based dosing is suitable for obese patients. Aiming for peak levels between 0.6 and 1.0 IU/mL in these patients would result in overexposure compared to controls. Considering the association of trough levels and bleeding risk and our findings, trough monitoring seems to be a suitable parameter to identify nadroparin accumulation.

## Introduction

Therapeutic doses of the low molecular weight heparin (LMWH) nadroparin are often used for peri-operative bridging in patients with atrial fibrillation or venous thromboembolism [1, 2]. Bridging anticoagulation consists of the substitution of a long-acting anticoagulant (usually a vitamin K antagonist or a direct oral anticoagulant (DOAC)) for a shorter-acting LMWH anticoagulant in order to reduce the (peri-operative) bleeding and thromboembolic risk during hospital admission. LMWHs are dosed on patient’s body weight and, unlike unfractionated heparin (UFH), LMWHs do not require routine monitoring of haemostatic parameters [1, 3–5]. However, for specific patient populations, such as patients with renal impairment or obesity, LMWH-dose adjustments and/or anti-Xa peak level monitoring is recommended in guidelines [5–7]. Since an analytical laboratory method for LMWH concentrations in blood is unavailable, anti-Xa levels are used as proxy for LMWH exposure [8].

Nadroparin is predominantly eliminated renally as unchanged drug, resulting in accumulation in patients with renal impairment [9, 10]. A meta-analysis showed elevated anti-Xa peak levels and increased bleeding risk in patients treated with unadjusted therapeutic doses of enoxaparin and a creatinine clearance below 30 mL/min. A pre-emptive dose adjustment was found to correct for both increasements [11, 12]. In this perspective, the Dutch Federation of Nephrology (NfN) recommends a pre-emptive dose adjustment with subsequent anti-Xa peak level monitoring for patients with renal impairment (i.e. creatinine clearance below 60 mL/min) receiving therapeutic doses of a LMWH, aiming for peak levels of 0.6 to 1.0 IU/mL for twice-daily dosed regimens [7]. Also, the American College of Chest Physicians Evidence-Based Clinical Practice Guidelines on antithrombotic therapy states that dose reduction and/or subsequent anti-Xa monitoring should be considered in patients with a creatinine clearance below 30 mL/min [5]. On the other hand, the ASH guideline panel advices against the use of anti-Xa monitoring to guide LMWH dosing in patients with renal impairment or obesity, due to the weak correlation with bleeding events and concerns about anti-Xa test standardisation [13].

To date, there is only limited evidence for anti-Xa peak level monitoring, since a clear correlation with bleeding risk or antithrombotic efficacy in these patient populations is lacking [8]. Also from a pharmacokinetic point of view, monitoring peak levels as a proxy for drug accumulation of a solely renally excreted drug in patients with renal impairment seems to be less sensitive than monitoring trough levels [8, 14].

In morbidly obese patients, weight-based dosing (i.e. IU/kg) may lead to relatively high anti-Xa peak levels compared to non-obese patients, since the distribution volume increases in a non-linear manner with the patients total body weight (TBW) [15]. However, dose capping at a certain total body weight threshold may result in undertreatment of individuals with a total body weight far above the cut-off point. Clear evidence-based guidelines on LMWH dosing and monitoring in obese patients are currently lacking [5, 6, 16–18]. Given obesity-induced changes in both volume and clearance [19], both peak and trough levels may be altered.

In this prospective observational study, the effect of renal impairment and obesity on anti-Xa peak and trough levels is evaluated in patients receiving therapeutic doses of nadroparin with an anticipated treatment duration of at least three days. Besides a descriptive analysis of the results, population pharmacokinetic (pop PK) modelling was used to analyse the data in conjunction with a previously published rich dataset consisting of full anti-Xa curves in non-obese and morbidly obese individuals [19]. This combined model can be used to guide dose individualisation for patients with renal impairment or obesity.

## Methods

### Patient inclusion

Adult hospitalised patients treated with therapeutic doses of nadroparin (86 IU BID subcutaneously (SC), rounded to the nearest available formulation, or adjusted at the physician’s discretion) were screened for inclusion between August 2020 and January 2021.

Patients were excluded in case they had an active SARS-CoV-2 infection (PCR-confirmed), a congenital coagulation disorder, underwent haemodialysis, were admitted at the intensive care unit or in case the intended use of nadroparin treatment was shorter than three days. Moreover, patients that had a BMI of at least 30 kg/m^2^ combined with an eGFR below 50 mL/min were also excluded. The study protocol was approved by the Independent Ethics Committee [MEC-U, protocol ID NL71527.100.19], and written informed consent was signed by each participating patient.

During patient screening, subjects were included in four separate groups: (a) normal creatinine clearance (eGFR > 50 mL/min/1.73m^2^) with BMI below 30 kg/m^2^ (i.e. controls), (b) mild renal impairment (30–50 mL/min/1.73m^2^) with BMI below 30 kg/m^2^, (c) severe renal impairment (< 30 mL/min/1.73 m^2^) with BMI below 30 kg/m^2^, and (d) normal creatinine clearance but with a BMI of at least 30 kg/m^2^.

Local protocol prescribed subcutaneously nadroparin dosage strata of 3800 IU BID for body weights of < 60 kg, 5700 IU BID for 60–80 kg, 7800 IU BID for 80–100 kg, and 9500 IU BID for > 100 kg. Furthermore, a pre-emptive dose reduction to 75 and 50% of the original dose was prescribed for patients with mild and severe renal impairment respectively.

### Blood sampling and analysis

Blood was collected for measurement of anti-Xa peak and trough levels at steady state (LMWH treatment ≥ 72 h based on the elimination half-life). Protocol prescribed blood collection 30 min before and 4 h after nadroparin administration for anti-Xa trough and peak level determination respectively. Anti-Xa levels were classified as correctly drawn peak levels if collected 3 to 5 h after dosing, and as trough levels if collected 10 to 14 h after dosing. Blood collections and administration of nadroparin were performed according to daily routine with the nursing staff requested to register the exact time of administration and blood collection in the patient file. Blood samples were stored at room temperature and centrifuged as soon as possible (within 4 h after collection). Plasma levels of anti-Xa activity were measured with a STA-R Max Evolution system (Diagnostica Stago, Asnières, France) and corresponding reagents (Liquid anti-Xa, Diagnostica Stago, Asnières, France) (calibration curve 0.0–1.6 IU/mL, error 3.2%).

### Data analysis

Anti-Xa levels were evaluated in a descriptive manner using Microsoft Excel (2016). Anti-Xa peak and trough levels outside the 0.6–1.0 IU/mL and < 0.5 IU/mL range were identified respectively. Creatinine clearance (using the CKD-EPI formula) was expressed both indexed and deindexed for BSA.

For the popPK analysis, all anti-Xa data (peak, trough, and in-between levels) of the current study were added to the previously published rich dataset of anti-Xa data of twenty-eight morbidly obese and seven non-obese patients with normal creatinine clearance receiving a single dose of 5700 IU and 2850 IU SC nadroparin before surgery, respectively. In the latter study, the mean total body weight was 135 kg (range 72–252 kg) and up to 11 anti-Xa levels were collected per patient (with the earliest collections before start of nadroparin, and the latest collections at 24 h after nadroparin administration) [19].

The combined dataset was analysed using non-linear mixed effects modelling with NONMEM (version 7.4; ICON Development Solutions, MD, USA) and the Pirana interface (v2.9.9). R (v3.5.3). Rstudio (v1.1.463) and Xpose (v4.6.1) were used to evaluate and visualise the data. Discrimination between different models was guided by comparison of the objective function value (OFV, i.e. − 2 log likelihood (− 2LL)). A *p* value of < 0.05, representing a decrease of 3.84 in OFV for one degree of freedom, was considered statistically significant. In addition, goodness-of-fit plots (observed vs. individual-predicted anti-Xa level, observed vs. population-predicted anti-Xa level, conditional weighted residuals vs. time after dose, conditional weighted residuals vs. time, and conditional weighted residuals vs. population-predicted anti-Xa level plots) of all data and split per study cohort were used for diagnostic purposes. Furthermore, precision of parameter estimates, the correlation matrix and visual improvement in the individual plots were used to evaluate the model. A one-compartment model and a two-compartment model with one and two transit compartments were evaluated. As in the current study no baseline endogenous anti-Xa levels were collected, the baseline anti-Xa level was fixed to the population estimate (i.e. 0.021 IE/mL) [19]. The transit rate constant (Ktr) was set equal to the absorption rate constant (Ka) resulting in similar performance of the model. Interindividual variability (IIV) was assumed to follow a log-normal distribution. Residual variability was described with an additive error model. For internal model evaluation, a bootstrap resampling method stratified for study using 250 replicates was used.

In the covariate analysis, covariates were plotted independently against the individual estimates of the pharmacokinetic parameters to visualise potential relations. The following covariates were tested: total body weight, body mass index (BMI), lean body weight (LBW) (see formulas 1 and 2), age, sex, creatinine, body surface area (BSA), and estimated glomerular filtration rate (Chronic Kidney Disease Epidemiology Collaboration (CKD-EPI)). The CKD-EPI equation provides an eGFR for a standardised body surface area of 1.73 m^2^ (i.e. mL/min/1.73 m^2^). eGFR values were deindexed by multiplying the CKD-EPI value with the individual BSA (Du Bois). Covariates were implemented in the model using forward inclusion (*p* < 0.005, OFV decrease > 7.8) and backward deletion (*p* < 0.001, OFV decrease 10.8) Furthermore, the contribution of a covariate was judged based on the reduction in IIV and diagnostics.

1 $$\mathrm{Lean\; body\; weight\; male\; (kg)} = \frac{9270\times \mathrm{TBW}}{6680+216\times \mathrm{BMI}}$$

2 $$\mathrm{Lean\; body\; weight\; female\; (kg)} = \frac{9270\times \mathrm{TBW}}{8780+244\times \mathrm{BMI}}$$

Model-based evaluations were performed using the final model in which the impact of renal impairment, and obesity on anti-Xa levels was visualised upon weight-based dosing with and without dose reductions for renal impairment in three representative male and female non-obese patients with varying creatinine clearance (i.e. eGFR 90 mL/min/1.73 m^2^, eGFR 40 mL/min/1.73 m^2^, and eGFR 25 mL/min/1.73m^2^; total body weight 70 kg and 80 kg for female and male patients, respectively, with corresponding lean body weights of 44 and 62 kg) and one representative female and male obese patient with normal creatinine clearance (eGFR 90 mL/min/1.73 m^2^, total body weight 95 and 105 kg, BMI 32.1, and 31.7 kg/m^2^ for female and male patients respectively, with corresponding lean body weights of 53 and 72 kg). Only nadroparin dosages of commercially available formulations were used in the model-based evaluation (e.g. 3800 IU, 5700 IU, and 7600 IU), resulting in slightly varying dose reductions between representative patients with renal impairment (to 75% and 67% of original dose for male and female patients with mild renal impairment respectively, see Fig. 3B).

## Results

Seventy-six patients were screened, from whom 23 were excluded. Of the eligible 53 patients, 27 consented to participate. The renal impairment and obesity subgroups each comprised 5 patients and the control group comprised 12 patients. In total, 68 anti-Xa levels were available of which 17 (25%) were classified as correctly drawn peak levels and 20 (29%) as correctly drawn trough levels. All 68 anti-Xa levels were used in the pop PK analysis. The baseline patient characteristics are provided in Table 2.

**Table 1:** Patient characteristics, nadroparin dosages, and anti-Xa measurements

	**Control group** **(BMI < 30, eGFR > 50)**	**eGFR 30–50** **(BMI < 30, eGFR 30–50)**	**eGFR < 30** **(BMI < 30, eGFR < 30)**	**Obese patients** **(BMI ≥ 30, eGFR > 50)**
**Demographics**				
***n***	12	5	5	5
**Age (years)**	72 (49–79)	70 (65–81)	75 (65–89)	64 (38–87)
**Female(%)**	25	20	20	40
Total body weight (kg)*	71 (52–110)	81 (61–95)	78 (73–90)	101 (87–103)
**BMI (kg/m**^**2**^)*	23 (18–30)	27 (20–30)	27 (25–29)	34 (30–35)
Lean body weight (kg)*	58 (36–78)	61 (51–68)	60 (56–65)	66 (54–70)
**eGFR (mL/min/1.73m**^**2**^)*	90 (44–104)	35 (27–48)	27 (24–30)	81 (53–129)
eGFR-deindexed (mL/min)*	97 (50–145)	41 (34–48)	30 (25–35)	89 (67–158)
**Nadroparin dosage**				
**Absolute dosage (IU/kg)**	89 (81–111)	70 (60–94)	38 (35–52)	92 (58–94)
**Relative dosage (percentage of non-adjusted dosage of 86 IU/kg)**	100 (100–125)	75 (75–100)	50 (38–66)	100 (75–100)
**Number of patients with adjusted dosage (*n*)**	0 /12	3 /5	5 /5	1 /5
**Anti-Xa measurements**				
Day of first anti-Xa measurement**	4 (2–29)	13 (6–19)	3 (2–14)	7 (6–11)
Day of second anti-Xa measurement**	6 (5–29)	14 (13–14)	N/A	9 (8–13)
**Median amount of anti-Xa levels per patient (*n*)**	2 (1–4) Peak: 0 (0–2) Trough: 1 (0–2)	2 (1–3) Peak: 1 (0–1) Trough: 1 (0–2)	2 (1–7) Peak 1 (0–2) Trough: 1 (0–1)	3 (1–4) Peak: 0 (0–2) Trough: 1 (0–2)
**Total of anti-Xa levels (*n*/#unique patients)**	Total: 29 Peak: 7/5 Trough: 8/6	Total: 10 Peak: 3/3 Trough: 5/4	Total: 15 Peak: 4/3 Trough: 3/3	Total: 14 Peak: 3/2 Trough: 4/3
**Peak anti-Xa level (IU/mL)** **Median (range)**	0.43 (0.34–0.82)	0.59 (0.52–1.06)	0.35 (0.22–0.83)	0.60 (0.26–0.95)
**Trough anti-Xa level (median) IU/mL (*n*)** **Median (range)**	0.29 (0.12–0.52)	0.35 (0.28–0.65)	0.41 (0.18–0.49)	0.53 (0.07–1.04)

Values are shown as medians (range). Anti-Xa peak and trough levels are defined as levels collected 3–5 h and 10–14 h after nadroparin administration respectively (with a minimal nadroparin treatment of 72 h). Lean body weight is calculated according to Eq. 1 and 2

*BMI* body mass index, *eGFR* estimated glomerular filtration rate calculated with the CKD-EPI formula with de-indexation using the BSA of the patient, *relative dosage of nadroparin (percentage)* percentage of the nadroparin dose as compared to weight based dosing (86 IE/kg), *number of patients with adjusted dosage* number of patients with a nadroparin dosage < 100%

*Demographics are collected at the day of the first anti-Xa measurement

**Day of anti-Xa measurement compared to the first day of nadroparin treatment

***When multiple anti-Xa peak or trough levels were measured for the same patient, the average of these levels was used. In the control group, 7 anti-Xa peak levels were measured in 5 patients

**Fig. 1:**
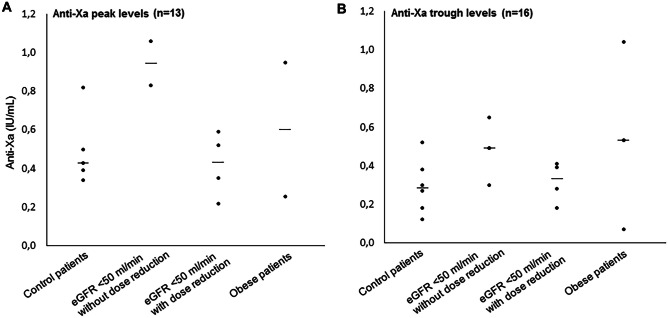
Observed anti-Xa levels. **A** Anti-Xa peak levels (*n* = 13). **B** Anti-Xa trough levels (*n* = 16). Values are shown as individual anti-Xa measurements (dot) with median (line). Measurements of patients with mild and severe renal impairment (eGFR < 50 mL/min) were merged into one group and subsequently subdivided into groups with and without nadroparin dose reduction. For patients with two anti-Xa peak or trough levels, the average peak or trough level is shown. eGFR: estimated glomerular filtration rate calculated with the CKD-EPI formula and deindexed for body surface area

Figure 1 shows the observed anti-Xa peak and trough levels. Due to the low number of correctly collected peak and trough levels, data from patients in the mild and severe renal impairment groups were merged. Observations in individuals with and without a pre-emptive dose reduction are presented separately. In the control group, median anti-Xa peak and trough levels were 0.43 IU/mL (range 0.34–0.82 IU/mL, *n* = 5) and 0.29 IU/mL (range 0.12–0.52 IU/mL, *n* = 6) respectively. In the control group, one (20%) anti-Xa peak level was within the therapeutic range of 0.6–1.0 IU/mL and five (83%) anti-Xa trough levels were below 0.5 IU/mL, and all were below 0.6 IU/mL. Mean anti-Xa peak and trough levels in patients with a creatinine clearance of < 50 mL/min with dose adjustment were comparable to the anti-Xa levels of the control patients (median anti-Xa peak 0.44 IU/mL, range 0.22–0.59 median anti-Xa trough 0.34 IU/mL, range 0.18–0.41). From the patients with available anti-Xa peak and trough levels, three patients with a creatinine clearance of < 50 mL/min did not receive a dose reduction. Their median anti-Xa peak and trough levels were 0.95 (range 0.83–1.06 IU/mL) and 0.49 IU/mL (range 0.3–0.65 IU/mL) respectively, which is higher than the median observations in patient with dose adjustments. The 5 obese patients had a median anti-Xa peak level of 0.60 IU/mL (range 0.26–0.95 IU/mL, *n* = 2) and a median trough level of 0.53 IU/mL (range 0.07–1.04 IU/mL, *n* = 3). The obese patients demonstrated relatively high inter-individual variability in anti-Xa trough levels, resulting in a relatively high median anti-Xa trough level, mostly due to one high trough level of 1.04 IU/mL.

We observed anti-Xa peak levels which were lower than anti-Xa trough levels in three patients. In all cases, the anti-Xa trough level was reported to be collected before the morning administration of nadroparin and the anti-Xa peak level was collected 3–5 h after nadroparin administration on the same day.

In the pop PK analysis, all 68 obtained anti-Xa levels were jointly analysed with the previously published rich dataset of anti-Xa data of morbidly obese and non-obese patients with normal creatinine clearance [19]. A two-compartment model with a single transit compartment parametrized in ADVAN 5 adequately described the data. A two-compartment model was superior over a one-compartment model, resulting in better diagnostic plots. Similar to the original publication [19], the peripheral volume was set equal to the central compartment volume for precision estimation of the structural parameters. The pharmacokinetic parameters of the structural model without covariates are shown in Table 1. Because of the sparse data in the current study, shrinkage values of 38% and 40% were accepted for the volume of distribution and for the transit rate constant (Ktr).

**Table 2:** Population pharmacokinetic parameters of the base model without covariates and the final pharmacokinetic model for nadroparin and results of the bootstrap analysis median and 95% CI. *n* = 250 runs, 222 successful runs, inclusive significant digits < 3.0 errors, stratified on study

Parameter	Base model (RSE%) [shrinkage %]	Final model (RSE%) [shrinkage %]	Bootstrap (95% CI)
CL/*F* (mL/min)	30.7 (6.4)		
CL/*F* = CL/*F*_*pop*_ $\times \left(\frac{\mathrm{TBW}}{70}\right)\times \left(\frac{\mathrm{CKD}-\mathrm{EPI}}{90}\right)$ ^p^		21.0 (4.3)	21.0 (19.0, 23.2)
Exponent CKD-EPI (*p*)		0.46 (1.8)	0.45 (0.25, 0.64)
*V*_1_/*F* (mL)	9180 (13.5)		
*V*_1_/*F* = *V*_1_/*F*_*pop*_ $\times \left(\frac{\mathrm{LBW}}{60}\right)$ (mL)		8240 (8.1)	8289 (7482, 9195)
*V*_2_/*F* = *V*_1_/*F* (mL)			
*Q*/*F* (mL/min)	158 (22.1)	157 (7.8)	155 (124, 204)
*K*_*a*_ = *K*_*tr*_ (min^−1^)	0.0158 (7.1)	0.0157 (6.3)	0.0156 (0.0139, 0.0176)
BSL anti-Xa (IU/mL) in individuals of Diepstraten et al. [19]	0.0208 (6)	0.0226 (12.2)	0.0227 (0.0161, 0.0299)
BSL (aXa IU/mL) in individuals of current study	0.021 (fixed)	0.021 (fixed)	0.021 (fixed)
OFV (− 2LL)	-2021.674	-2082.617	-2095.450 (-2179.234, -2020.371)
Interindividual variability (*ω*^2^)			
CL/*F*	0.371 (74) [9]	0.115 (26) [22]	0.106 (0.0595, 0.166)
*V*_1_/*F*	0.0931 (185) [37]	0.0566 (39) [40]	0.0534 (0.0248, 0.0817)
Ktr = Ka	0.0725 (54) [37]	0.0576 (82) [38]	0.0525 (0.0217, 0.0907)
BSL anti-Xa	0.9 (42) [41]	0.763 (42) [39]	0.753 (0.439,1.36)
Residual variability (*σ*^2^)			
Additive error in individuals of Diepstraten et al. [19]	0.00043 (19)	0.00044 (14)	0.00044 (0.00035, 0.00056)
Additive error in individuals of current study	0.0252 (119)	0.0237 (3)	0.0229 (0.0141, 0.0370)

*CI* confidence interval, *CL* clearance, *F* bioavailability, *RSE* relative standard error, *Q* compartmental clearance between central compartment and peripheral compartment, *LL* log-likelihood, *V1* central volume of distribution, *V2* peripheral volume of distribution, *TBW* total body weight, *LBW* lean body weight, *OFV* objective function value, *BSL* baseline, *Ka* absorption rate constant, *Ktr* transit rate constant, *F* bioavailability.

**Fig. 2:**
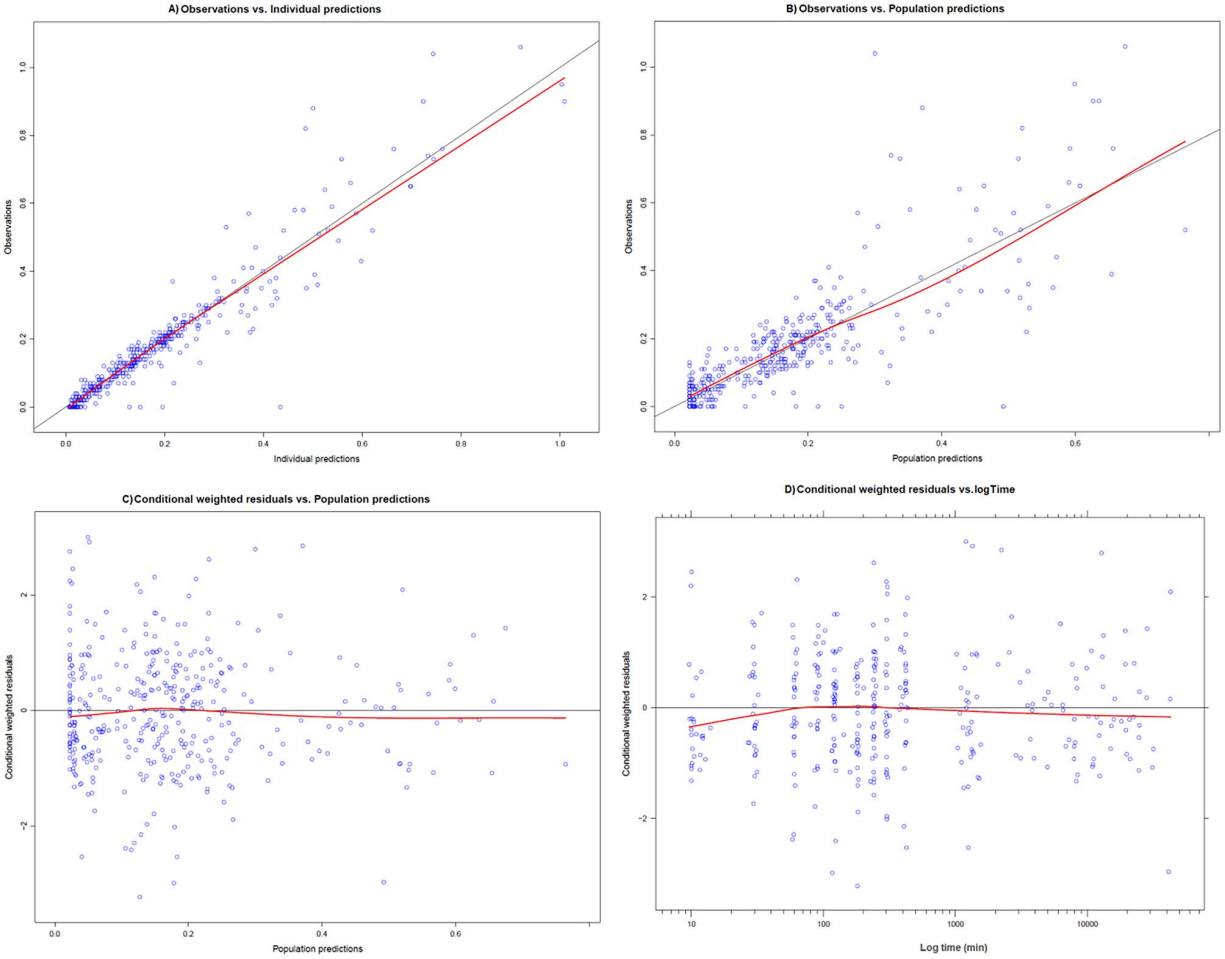
Diagnostic plots for the final model. **A** Observations (anti-Xa IU/mL) vs. individual anti-Xa level predictions (IU/mL), **B** observations vs. population anti Xa level predictions (IU/ml), **C** conditional weighted residuals versus population predictions, **D** conditional weighted residuals versus log time (time from start nadroparin (minutes)) of data of the current study together with previously reported data from Diepstraten et al. [19]. The solid line represents the line of identity, *x* = *y*; different symbols are used for different datasets

In the covariate analysis, total body weight and eGFR (deindexed CKD-EPI) proved the most significant covariates for clearance based on improvement in objective function of − 34.999 and − 33.026, respectively (both *p* < 0.001) (Table 1). The most optimal fit for the model was with the combination of total body weight (TBW) and CKD-EPI as covariates for clearance (total decrease in objection function of − 48.396, *p* < 0.001). For this model, no additional improvements in model fit or objective function value was observed by deindexing the CKD-EPI value with BSA, or implementation of other covariates like LBW. Adding lean body weight (LBW) on V1/F further decreased the objective function value (− 12.547). Figure 2 shows the goodness-of-fit plots of the final model, and Table 1 shows the model parameters of the final model.

**Fig. 3:**
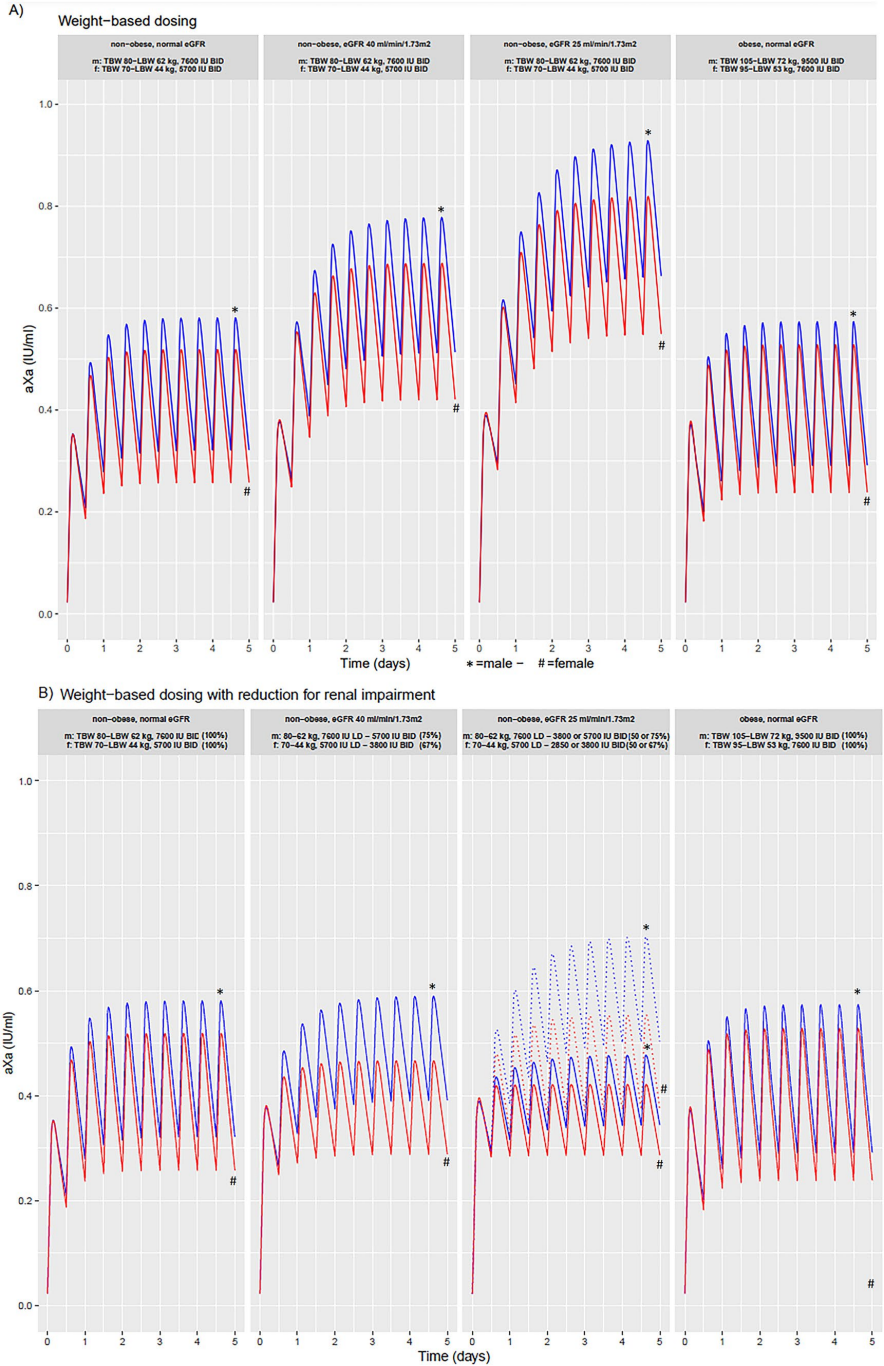
Model based evaluations of anti-Xa levels after **A** weight-based therapeutic dosing of nadroparin and **B** weight-based dosing with dose adjustment based on renal impairment in a three representative male and female non-obese patients (three left panels) and one representative male and female obese patient with normal creatinine clearance (right panel). #: female, *: male. Solid lines: 100% loading dose (LD), followed by 100% of the original dose for normal renal function, 67–75% for eGFR 40 mL/min/1.73 m^2^ and 50% for eGFR 25 mL/min/1.73m^2^. Dotted line: 100% LD, followed by 67–75% of the original dose for eGFR 25 mL/min/1.73 m^2^

Based on the final model, the impact of renal impairment and obesity on the anti-Xa level time profiles in relation to the nadroparin dose are visualised for different representative patients in Fig. 3. Figure 3A shows anti-Xa level-time profiles for control patients, patients with mild and severe renal impairment, and patients with obesity with a weight-based nadroparin dose of 86 IU/kg, rounded to the nearest available formulation. Both representative male and female patients are shown in the different groups to illustrate the influence of the lean body weight on the volume of distribution.

The figure shows subtherapeutic mean anti-Xa peak levels (< 0.6 IU/mL) for control patients (left panels). Obese patients (with a BMI of 32 kg/m^2^) had similar peak and trough levels upon weight based dosing compared to the control patients (right panels). For patients with renal impairment, higher peak and trough levels are observed. Figure 3B demonstrates that similar trough levels are obtained by dose adjustment to 75% of the original dose (rounded to the available formulations) for patients with mild renal impairment and to 50% for patients with severe renal impairment, although this results in lower peak levels. Dose adjustment to 67% of the original dose for patients with severe renal impairment results in similar peak levels but higher trough levels compared to the control patients.

## Discussion

In this study, anti-Xa peak and trough levels from patients with and without renal impairment or obesity obtained after three days of treatment were both evaluated descriptively and analysed combined with a previous published dataset of anti-Xa levels in obese and non-obese patients without renal impairment in a pop PK analysis. Patients with renal impairment with pre-emptive dose reductions had anti-Xa peak and trough levels similar to control patients. Patients without dose reductions had higher anti-Xa peak and trough levels. Obese patients receiving weight based dosing had similar anti-Xa levels to control patients.

## Anti-Xa levels in patients with renal impairment

Both the observational data and the pop PK analysis demonstrated elevated anti-Xa peak and trough levels in patients with renal impairment without pre-emptive nadroparin dose reduction. Patients with renal impairment with nadroparin dose reduction demonstrated anti-Xa levels similar to control patients. In the pop PK analysis, nadroparin dose reductions demonstrated a different effect on anti-Xa peak and trough levels. In patients with severe renal impairment a dose reduction to 50% of the original dose results in similar trough levels, but a dose reduction to 66% of the original dose results in similar peak levels compared to control patients. For patients with mild renal impairment a dose reduction to 75% of the original dose results in similar peak as well as a similar trough levels compared the controls (Fig. 3). Our results are in line with findings from a meta-analysis, which demonstrated elevated anti-Xa peak levels in patients with renal impairment receiving unadjusted therapeutic enoxaparin dosages. Patients with pre-emptive dose reductions had anti-Xa peak levels similar to control patients [12]. Also, van Ojik et al. demonstrated similar anti-Xa peak levels in patients with renal impairment compared to control patients when nadroparin dosage was reduced to 75% and 50% in patients with mild and severe renal impairment, respectively [20].

Based on these observations, routinely anti-Xa monitoring would be unnecessary in patients with renal impairment after adequate correction for pharmacokinetic differences [8]. Aiming in these patients at peak levels of 0.6–1.0 IU/mL would result in overexposure compared to patients without renal insufficiency.

## Anti-Xa levels in patients with obesity

In patients with obesity, sparse observational data on anti-Xa peak and trough levels demonstrated relatively high variability in anti-Xa peak and trough levels. In the combined pop PK model, evaluations in obese patients dosed on total bodyweight showed comparable peak and trough levels to non-obese control patients. These results include patients with BMI values up to 32 kg/m^2^ and are confirmed in previous results on LMWH therapy in obese patients with BMI values of up to 79 kg/m^2^ [19]. Since the LMWH volume of distribution increases proportionally to lean body weight in morbidly obese patients as observed in the final popPK model [19], dose capping may be required for extreme body weights in total body weight regimen. The relevance of dosing on lean body weight or total body weight in extremely obese patients (BMI > 50) may require further study.

## Anti-Xa levels in control patients

In control patients, the assumed therapeutic range for anti-Xa peak levels for twice daily dosed LMWHs (0.6–1.0 IU/mL) was only reached in one out of five (20%) control patients. Correspondingly, the pop PK analysis demonstrated anti-Xa peak levels below 0.6 IU/mL for simulated control patients with normal nadroparin dosages. Although this therapeutic range is used in daily care, previous publications confirm our findings of less than 50% of patients with neither renal impairment nor obesity achieving peak levels within this range [8, 12, 20–24]. Similar results were found in a more recent study of Hornung et al. in patients with renal impairment [25], in which 38% of anti-Xa peak levels were below this range. From these results, it seems that pursuing this range in special patient populations could result in higher anti-Xa exposure in these patients compared to control patients who do not require routine monitoring of their anti-Xa levels. Finally, our anti-Xa peak levels were lower than reported van Ojik et al., who demonstrated an average anti-Xa peak level of 0.62 IU/mL in control patients on therapeutic nadroparin [20]. This indicates differences in anti-Xa levels between different hospitals.

## Anti-Xa peak versus trough level monitoring

Although current guidelines suggest anti-Xa peak level monitoring in special patient populations [5–7], a clear correlation between peak levels and bleeding risk has not been demonstrated in patients with atrial fibrillation or venous thromboembolism. Therefore, the ASH guideline panel advices against the use of routine anti-Xa monitoring in special patient populations [13]. In contrast, anti-Xa trough levels above 0.5 IU/mL were reported to result in a doubling of the bleeding risk [26] and though levels are a respected parameter for drug accumulation in patients with reduced drug clearance [8, 14]. Anti-Xa trough levels were below 0.5 IU/mL in five out of six patients (83%) in our study. These findings were confirmed by our popPK model evaluation, which demonstrated anti-Xa trough levels below 0.5 IU/mL for simulated control patients with normal nadroparin dosages.

Considering the association of anti-Xa trough levels and bleeding risk and our findings, in which 4 out of 5 control patients had anti-Xa peak levels below the suggested range, whilst 5 out of 6 anti-Xa trough levels were below the 0.5 IU/mL threshold for accumulation, anti-Xa trough monitoring seems to be a suitable parameter to identify nadroparin accumulation [8].

## Variation in anti-Xa levels

A large variation in anti-Xa levels was observed both in the observational data and in the popPK analysis. This confirms previous publications [9, 10, 12, 17, 18, 21, 22, 27]. Using the developed pop PK model, an important part of the variation in anti-Xa levels could be explained by covariates such as total body weight and creatinine clearance, which already serve as important guidance for nadroparin dosage in daily practice. Residual variation may in part be caused by errors in registration of nadroparin administration or anti-Xa blood withdrawal since nadroparin administration and blood collections were performed in accordance with daily routine. Monitoring peak anti-Xa levels is a complex clinical challenge; two observational studies demonstrated a substantial number of anti-Xa blood collections were drawn at erroneous times [28, 29]. In our study, only 25% of all peak anti-Xa blood collections were drawn at the correct time. In this perspective, anti-Xa trough level monitoring could be less complex since the medical staff is used to obtain trough levels, which are required for the majority of drugs, and trough levels are less sensitive to the sampling time.

## Strengths and limitations

This study has several strengths and limitations. Our study provides information on anti-Xa peak and trough levels in control patients versus special populations such as patients with renal impairment and obesity. Blood collection and administration time were performed conform daily routine; therefore, we had to rely on the notes regarding administration and blood withdrawal in the electronic patient records, even in the case of the unexpectedly high value in obese patients. A relatively large number of anti-Xa levels could not be defined as peak or trough levels but was of important value for the pop PK analysis. By combining data with a previous published study, the influence of renal impairment and total body weight on anti-Xa levels was studied. Even though a relatively small number of patients was included in our study, solid conclusions can be drawn since these data are combined with the pop PK model and our findings confirm previous findings.

## Conclusion

In patients with renal impairment, dose reductions up to 50% are required, depending on whether similar anti-Xa peak or trough levels are pursued, to attain comparable anti-Xa levels as in controls. In obese patients, weight-based dosing is suitable, although dose capping should be considered for patients with extreme obesity. The majority of control patients have anti-Xa peak levels below the therapeutic window of 0.6–1.0 IU/mL. This implies that adjusting doses in special patient populations in order to attain anti-Xa peak levels within this range would result in overtreatment of these patients. Since dose reductions adequately correct for pharmacokinetic alterations in these patients, routinely monitoring of anti-Xa would no longer be necessary. However, in case anti-Xa monitoring is deemed necessary, monitoring of anti-Xa trough levels as a marker for drug accumulation could be considered.

## Data Availability

The data that support the findings of this study are available from the corresponding author, M van den Broek, upon reasonable request.
